# Case Report: Probable Myocarditis After Covid-19 mRNA Vaccine in a Patient With Arrhythmogenic Left Ventricular Cardiomyopathy

**DOI:** 10.3389/fcvm.2021.759119

**Published:** 2021-10-12

**Authors:** Edgardo Alania-Torres, Herminio Morillas-Climent, Alexandre García-Escrivá, Paul Vinueza-Buitrón, Inmaculada Poquet-Catalá, Esther Zorio, Ignacio José Sánchez-Lázaro, Emilio Galcerá-Jornet, Alfonso Valle-Muñoz

**Affiliations:** ^1^Cardiology Department, Marina-Salud Hospital, Denia, Spain; ^2^Neurology Department, Marina-Salud Hospital, Denia, Spain; ^3^Internal Medicine Department, Marina-Salud Hospital, Denia, Spain; ^4^Cardiology Department, La Fe University Hospital, Valencia, Spain

**Keywords:** myocarditis, vaccine, COVID-19, arrhythmogenic left ventricular cardiomyopathy, myopathy

## Abstract

Arrhythmogenic left ventricular cardiomyopathy (ALVC) is a rare heritable heart-muscle disorder characterized by a progressive loss of left ventricular myocardium and its replacement by fibrofatty tissue. Myocarditis is an inflammatory disease of the heart that may occur secondary to infections, immune system activation or exposure to drugs. Hot phases of ALVC present with chest pain and troponin rise, mimicking acute viral myocarditis and indicate a progression of the disease. Recently, myocarditis has also been described as an infrequent complication of coronavirus disease 2019 (Covid-19) mRNA vaccines. We herein report for the first time a case of probable myocarditis induced by Covid-19 vaccine in a patient with previous medical history of ALVC. We aim to highlight the common characteristics of ALVC and Covid-19 vaccine myocarditis and work through the differential diagnosis of these two entities.

## Introduction

The Covid-19 disease represents the largest worldwide health care challenge to date. Pfizer-BioNTech and Moderna Covid-19 vaccines (both mRNA) have significantly contributed to get the pandemic under control (1). The benefit-risk assessment for Covid-19 vaccination shows a favorable balance for all age and sex groups. However, there are a number of side-effects described after vaccination, including rare cases of myocarditis; according to the U.S. Centers for Disease Control (CDC), there are approximately 12.6 cases per million patients who receive a second dose among 12–39 year-olds (2).

ALVC is a variant of arrhythmogenic right ventricular cardiomyopathy in which the left ventricle is predominantly involved. The distinctive histopathological feature of ALVC is the loss of left ventricular myocardium, substituted by fibrous and fatty tissue. The natural history of ALVC may interpolate periods of clinical quiescence with hot phases, the latter being difficult to distinguish from a viral myocarditis (3).

Here, we report for the first time, to our knowledge, a case of probable myocarditis and associated myopathy induced by Covid-19 vaccine (BNT162b2 mRNA, Pfizer-BioNTech) in a young male patient previously diagnosed with ALVC.

## Case Description

A 28-year-old male patient was diagnosed of ALVC in 2012. He carried a heterozygous radical mutation in the desmoplakin gene (p.Gln1804^*^), just like his father. An implantable cardiac defibrillator (ICD) was inserted in 2013 for primary prevention of sudden cardiac death.

The patient possibly suffered from a hot phase of ALVC in 2009. In fact, he had 2 myocarditis-like episodes within 6 months. A comprehensive study including viral serologies was done, with no findings. The patient had a new episode of myocarditis in September 2020. There were no red flags that suggested a secondary cause and, due to background history, no specific aetiological study was performed. He never presented concomitant neuromuscular symptoms.

The patient overall performance was good, in a New York Heart Association I class, although his left ventricular ejection fraction (LVEF) was proved to be reduced down to 40% at last examinations, so his optimized medical treatment for heart failure with depressed ejection fraction included sacubitril/valsartan 24/26 mg bid, eplerenone 25 mg od and bisoprolol 1.25 mg od. He had no prior history of severe acute respiratory syndrome coronavirus 2 (SARS CoV-2) infection.

In January 2021, the patient received the second dose of the Covid-19 vaccine (BNT162b2 mRNA, Pfizer-BioNTech). The next morning, he developed fever, myalgias, weakness, headache and diarrhea. Two weeks later, he was admitted to the hospital with progressive muscle weakness involving the scapular and pelvic girdles, chest pain, fatigue and dyspnea.

At admission, blood pressure was 137/74 mmHg, heart rate was 77 beats/min, temperature was 36°C and a normal blood oxygen level was confirmed. Physical examination did not show cardiac murmurs, rubs nor pulmonary crackles. The neurological assessment confirmed proximal muscle weakness, mainly localized at the lower limbs. Muscle stretch reflexes were mildly and symmetrically diminished. The patient could stand up, but presented a plodding, unstable gait and shuffling due to lack of muscle strength.

**Figure 1:**
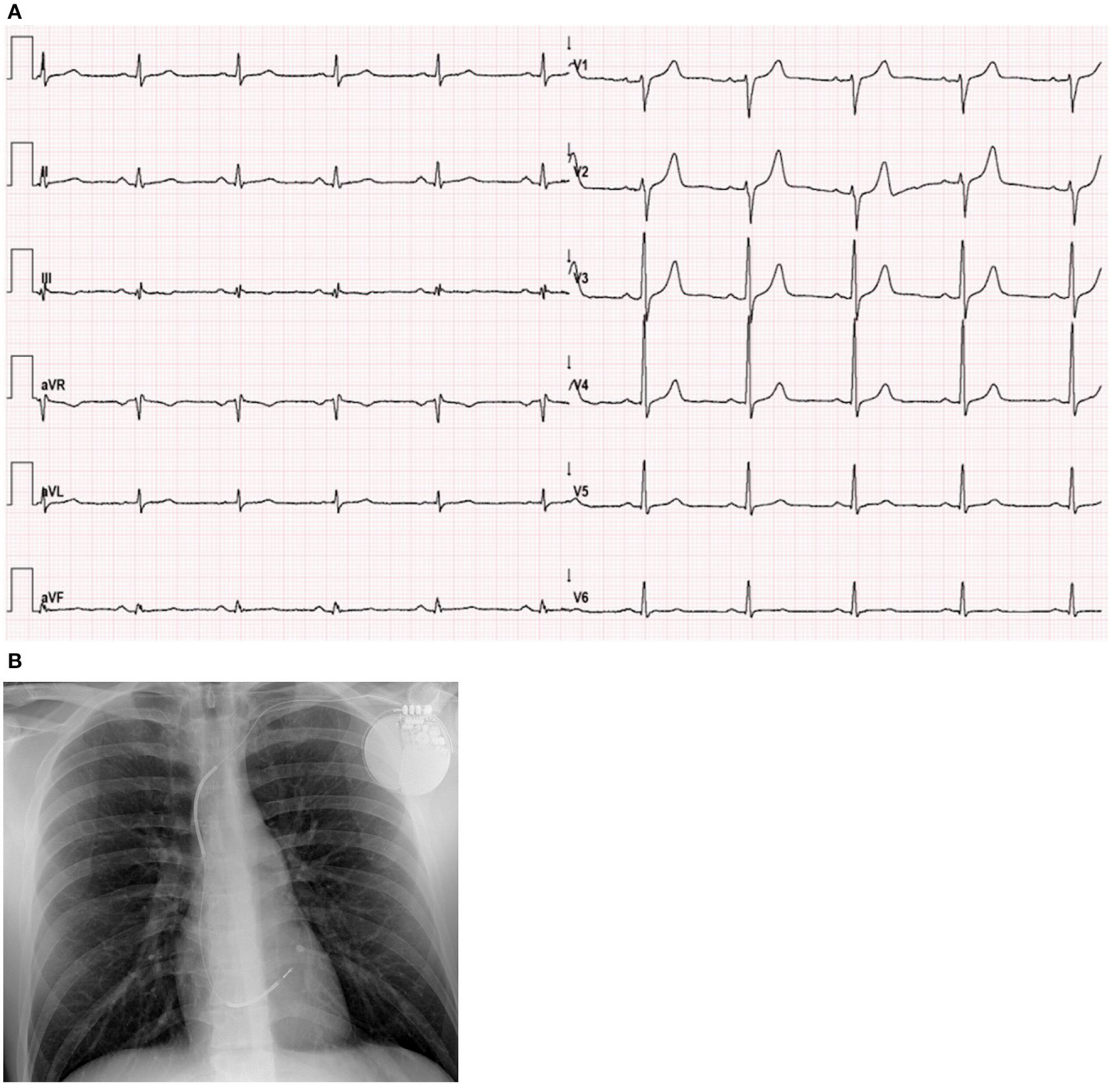
Electrocardiogram and Chest X-ray. **(A)** ECG showed sinus rhythm, 60 bpm, normal axis, fragmented QRS in the inferior leads (III, aVF) and flat T waves in III, aVF and V6. When compared to previous ECGs, no new abnormalities were observed. **(B)** Chest X-ray with a single lead ICD, normal heart size and no signs of pulmonary congestion.

**Figure 2:**
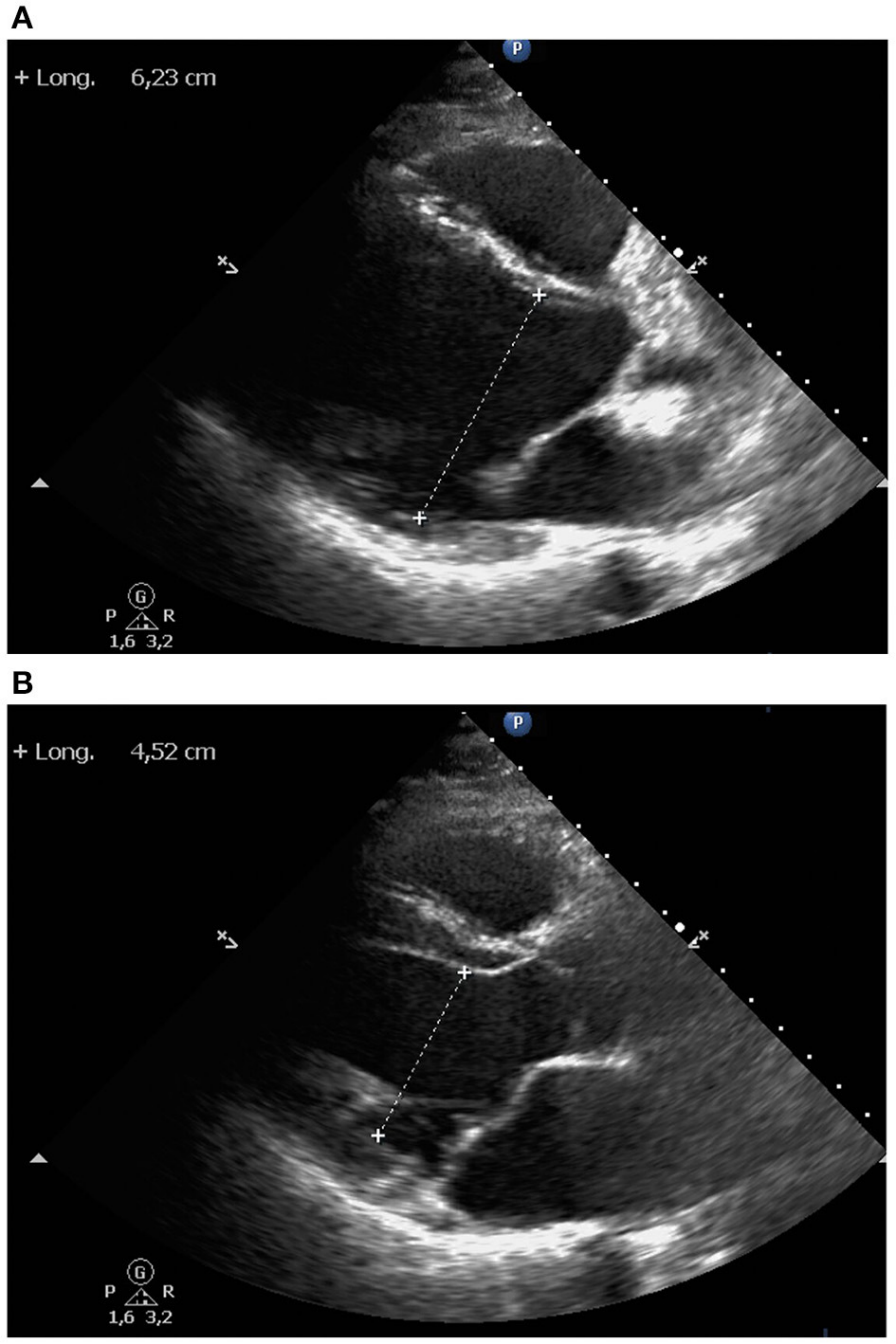
Echocardiography. Parasternal long axis view showed mild left ventricular enlargement, both left ventricular end-diastolic **(A)** and end-systolic **(B)** diameters. Right ventricle, ascending aorta and left atrium were of normal size. Interventricular septum and posterior left ventricular wall had normal thickness. No pericardial effusion was observed.

Initial evaluation at the hospital revealed a negative SARS-CoV-2 polymerase chain reaction test result. His electrocardiogram (ECG) showed sinus rhythm with fragmented QRS and flat T waves in the inferior leads; no new abnormalities were identified when compared to previous ECG. There were no signs of heart failure in the chest X-ray (Figure 1). At admission, the high sensitivity cardiac troponin I (hs-cTnI) level was 5,052 ng/l (normal range 0–58.05 ng/l). N-terminal pro–B-type natriuretic peptide level was 89 pg/ml (normal range 0–100 pg/ml). Creatine kinase (CK) was slightly increased, both the total and isoenzyme MB levels (271 IU/l and 15.77 ng/ml, respectively). The rest of laboratory tests remained unremarkable, without abnormal acute inflammatory markers (C reactive protein <0.5 mg/l). No cytokine measurements nor immunofluorescence tests were carried out. The echocardiographic assessment showed mild left ventricular enlargement (Figure 2) with moderate left ventricular systolic dysfunction (Supplementary Videos 1,2). No changes from baseline were observed. No arrhythmic events were detected during ICD interrogation.

Neurological tests included a cranial computed tomography (CT) scan and a brain magnetic resonance imaging (MRI), which did not show any abnormalities. Cerebrospinal fluid analysis was normal: glucose and proteins levels were within normal range, leukocytes were undetectable and no oligoclonal bands were identified. The electroneurography/electromyography (ENG/EMG) conduction study during the initial acute phase revealed a moderate intensity myopathic pattern mostly affecting the lower limbs with difficulty in activating motor units. These alterations completely resolved in the follow-up ENG/EMG performed 5 days later. A bilateral muscular MRI showed appropriate thigh morphology and thickness, without abnormalities in gadolinium enhancement sequences. The autoimmunity panel was unremarkable.

**Figure 3:**
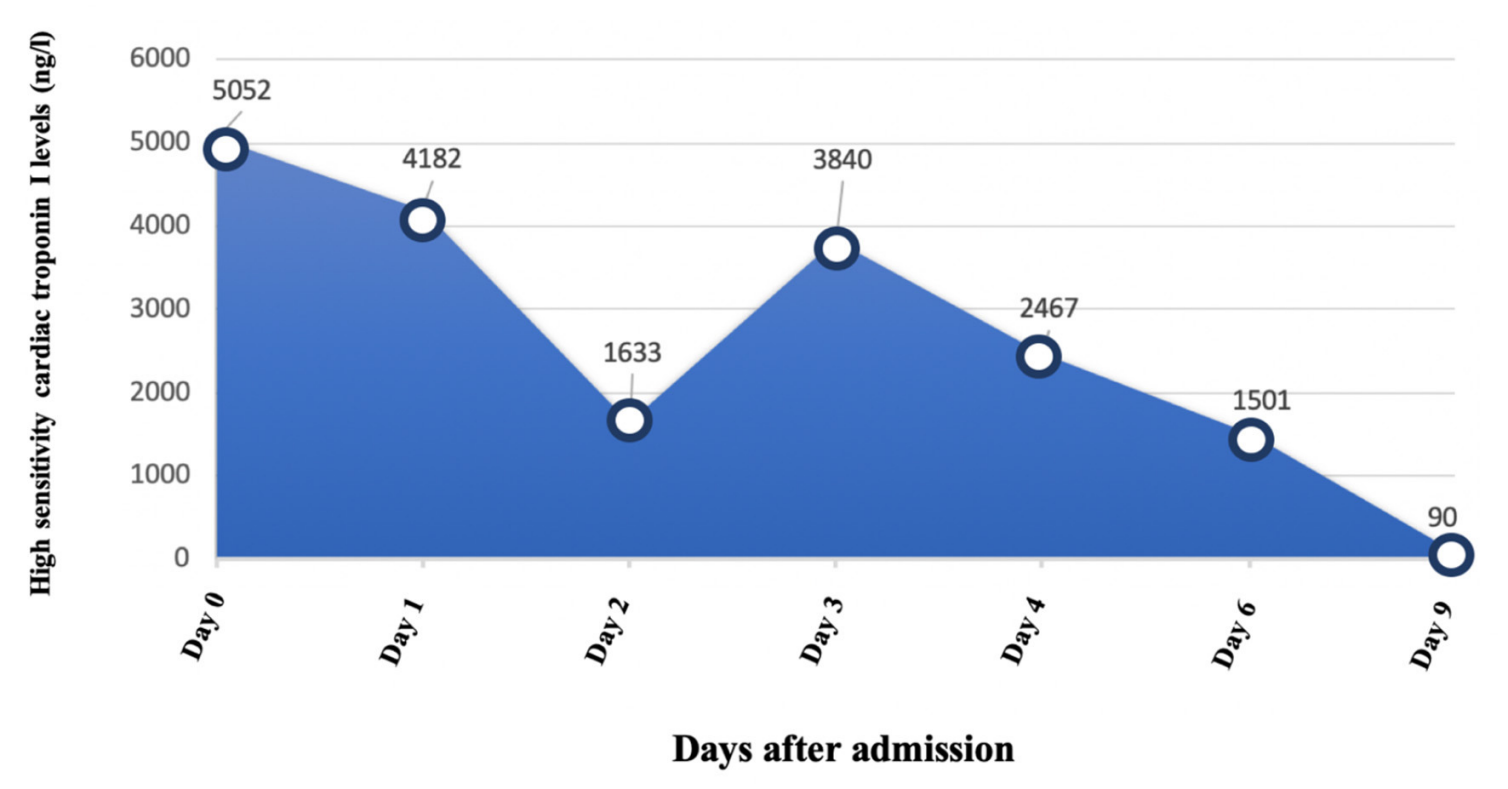
Dynamic of high sensitivity cardiac troponin I levels. Hs-cTnI levels peaked at admission. Three days later, with ongoing recurrent episodes of chest pain, a further increase in hs-cTnI value was observed. After starting aspirin and colchicine, chest pain resolved and hs-cTnI levels progressively decreased. At discharge, hs-cTnI value was almost normal (normal range 0–58.05 ng/l).

Patient was evaluated through a comprehensive and multidisciplinary approach by Cardiology, Neurology and Internal Medicine. Initial treatment included acetaminophen, metamizol and colchicine. However, during the first 3 days the patient required intravenous opioids to manage the severe headache, recurrent chest pain episodes and progressive muscular weakness. After ruling out alternative infectious agents and supported by the high suspicion of an immune-mediated etiology, intravenous steroids were suggested. Nonetheless, the patient refused to take them due to past secondary effects in the context of previous myocarditis events. At that point, aspirin was started and progressively uptitrated based upon clinical and biochemical improvement during previous myocarditis relapses and considering a significant pericardial involvement (1 g tid aspirin and 0.5 mg bid colchicine). After the fifth day of stay, symptoms progressively ameliorated and, 5 days later, the patient was discharged with mild muscle weakness and almost normal hs-cTnI levels (Figure 3).

During short-term follow-up, the patient did not present any episodes of chest pain nor dyspnea. Blood tests including N-terminal pro–B-type natriuretic peptide, CK, hs-cTnI and acute inflammatory markers were unremarkable. Two months later, echocardiography was repeated and showed a mild improvement of left ventricular systolic function (Supplementary Video 3).

## Timeline

**Table:** 

2012	Diagnose of ALVC.
September 2020	Last episode of myocarditis (without neurological symptoms).
January 2021	Administration of boost dose of mRNA Covid-19 vaccine.
February 2021 (14 days after boost dose)	Admitted for chest pain, elevated hs-cTnI and neurological symptoms.
First 3 days of admission	Recurrent chest pain despite treatment with acetaminophen, metamizol and colchicine. Stable moderate left ventricular systolic dysfunction without signs of heart failure Moderate intensity myopathic pattern in ENG / EMG
Day 4 of admission	Patient refuses to initiate steroids due to past secondary effects Aspirin is started and progressively uptitrated
5–9 days after admission	Gradual improvement of symptoms. Decline of hs-cTnI levels. Complete resolutions of abnormalities in the follow-up ENG/EMG.
Day 10 after admission	Patient is discharged

## Discussion

As far as we know, here we report the first suspected case of myocarditis and myopathy induced by Covid-19 vaccine in a patient with ALVC. Most of the reported systemic events of the Covid-19 vaccines were due to reactogenicity, with a typical onset within the first 24 h. These events were generally transient and resolved spontaneously. Recently, a significant number of myocarditis cases post Covid-19 vaccination have been published. The majority of the patients presented with chest pain, usually 2–3 days after the second dose of mRNA vaccination, and sometimes chest pain was preceded by fever and myalgia (4, 5).

We want to highlight some characteristics that Covid-19 vaccine-related myocarditis and ALVC have in common. First, age of onset of ALVC and vaccine-induced myocarditis are similar, ranging between 12 and 39 years.

Second, post-vaccine myocarditis has shown a male predominance, possibly related to testosterone levels, by a combined mechanism of inhibition of anti-inflammatory cells and commitment to a Th1-type immune response (6). At the same time, male patients have a three-fold-risk incidence of ALVC compared to females, develop the disease earlier and present more severe phenotypes and a higher arrhythmic risk. A possible explanation for the latter could lie in the differences of physical exercise between genders; one study found that elevated serum testosterone levels in males and decreased estradiol levels in females are independently associated with MACE (7, 8).

Third, autoantibody generation could be one of the mechanisms of myocarditis in susceptible individuals after vaccination (9). There is molecular mimicry between the spike protein of SARS CoV-2 and self-antigens, and antibodies against SARS CoV-2 spike glycoproteins have been experimentally shown to cross-react with structurally similar human peptide protein sequence, including alpha-myosin. It is possible that it may trigger preexisting dysregulated pathways in certain individuals with predisposition, resulting in a polyclonal B-cell expansion, immune complex formation and inflammation (10, 11). The presence of serum anti-heart autoantibodies and anti-intercalated disk autoantibodies provides evidence of autoimmunity in the majority of familial and in almost half of sporadic ALVC. Increasing evidence of autoimmunity has also been recently reported with two studies identifying anti-heart, anti-intercalated disc and anti-DSG2 autoantibodies in patients with ALVC (12, 13).

Regarding our case, the diagnosis of myocarditis was made in light of recurrent chest pain and hs-cTnI elevation without an alternative explanation. Notwithstanding, the differential diagnosis between myocarditis and a hot phase of the ALVC is challenging, since most of the ECG, echocardiographic and MRI findings may overlap and, moreover, the two of them might be pathophysiologically related. Hot phases of ALVC present with chest pain and troponin rise, mimicking acute viral myocarditis and indicate a progression of the disease (14). However, the severity of the hs-cTnI peak, the positive response to anti-inflammatory drugs and the subsequent improvement in LVEF 2 months later support the diagnosis of a new myocarditis episode. Between 10 and 25% of patients included in case series of myocarditis after Covid-19 vaccination had normal values of C reactive protein. Acute inflammatory markers could more frequently be within the normal range when the cause of myocarditis is not an infectious agent. Cardiac MRI adds very useful information to the working diagnosis of myocarditis. However, our patient carried an ICD, so we did not perform this test due to image-quality and safety issues. Definite diagnosis of myocarditis relies on endomyocardial biopsy, but this an invasive procedure associated with potentially life-threatening risks, and in the daily clinical practice it is limited to a selected number of complicated clinical scenarios (15).

Myopathy diagnosis was based upon muscular symptoms, an increase in total CK and an abnormal neurophysiological study result. Artifacts related to muscle weakness and pain could have blunted the interpretation of neurophysiological study. However, the increase in CK could not be justified by the isoenzyme MB alone and thus skeletal muscle must have been to some extent involved. A muscle biopsy was initially considered, but discarded afterwards due to potential risks and subsequent patient clinical improvement.

Temporal relationship and concomitant neuromuscular symptoms suggest the boost dose could have played a significant role in the genesis of myocarditis in this case. One possible hypothesis is that the spike protein of the mRNA vaccine could have developed antibodies that in genetically predisposed patients can attack cardiac muscle proteins (sarcomeric, desmosomal, nuclear), which eventually leads to the event of myocarditis. Further research should be designed to explore predisposing factors for development of myocardial injury (genetic factors, comorbidities or autoimmunity profile). Close monitoring and surveillance after boost dose of Covid-19 vaccine could be useful in ALVC patients.

## Data Availability Statement

The original contributions presented in the study are included in the article/Supplementary Material, further inquiries can be directed to the corresponding author/s.

## Ethics Statement

Written informed consent was obtained from the individual for the publication of any potentially identifiable images or data included in this article.

## Author Contributions

All authors contributed to the analysis and interpretation of data, wrote the manuscript, approved the final version of the manuscript, and agreed to be accountable for all aspects of the work.

## Conflict of Interest

The authors declare that the research was conducted in the absence of any commercial or financial relationships that could be construed as a potential conflict of interest.

## Publisher's Note

All claims expressed in this article are solely those of the authors and do not necessarily represent those of their affiliated organizations, or those of the publisher, the editors and the reviewers. Any product that may be evaluated in this article, or claim that may be made by its manufacturer, is not guaranteed or endorsed by the publisher.

## Supplementary Material

Supplementary Video 1 2D paraesternal long-axis displayed mild left ventricular dilation with moderate left ventricular systolic dysfunction. Mitral and aortic valve leaflets were thin and mobile, with good opening and closure. No pericardial effusion was observed.

Supplementary Video 2 2D apical four chamber showed mild left ventricular spherical remodeling, LVEF of 40% and absence of regional wall motion abnormalities. The right ventricle had normal size and systolic function. Both left and right atriums were not dilated.

Supplementary Video 3 2D apical four chamber 2 months after hospital discharge exhibited an elliptical-shaped left ventricle with a mild improvement in left ventricular systolic function. LVEF assessment by Simpson's method was 50%.

## Abbreviations

ALVC: arrhythmogenic left ventricular cardiomyopathy
Covid-19: coronavirus disease 2019
ICD: implantable cardiac defibrillator
LVEF: left ventricular ejection fraction
SARS CoV-2: severe acute respiratory syndrome coronavirus 2
ECG: electrocardiogram
hs-cTnI: high sensitivity cardiac troponin I
CK: creatin kinase
CT: computed tomography
MRI: magnetic resonance imaging
ENG/EMG: electroneurography/electromyography.
